# The role of opioid receptors in modulating Alzheimer’s Disease

**DOI:** 10.3389/fphar.2023.1056402

**Published:** 2023-03-01

**Authors:** Parthasaradhireddy Tanguturi, John M. Streicher

**Affiliations:** ^1^ Department of Pharmacology, College of Medicine, University of Arizona, Tucson, AZ, United States; ^2^ Comprehensive Pain and Addiction Center, University of Arizona, Tucson, AZ, United States

**Keywords:** neurodegenerative disorders, Alzheimer’s disease, opioid receptors, β-secretase-1, amyloid-β

## Abstract

Alzheimer’s disease (AD) is a complex neurological disorder characterized by accumulation of amyloid plaques and neurofibrillary tangles. Long term investigation of AD pathogenesis suggests that β-site amyloid precursor protein [APP] cleaving enzyme 1 (BACE1) and γ-secretase enzymes promote the amyloidogenic pathway and produce toxic Aβ peptides that are predisposed to aggregate in the brain. Hence, the targeted inhibition of BACE1/γ-secretase expression and function is a promising approach for AD therapy. Several reports have suggested that the opioid family of G-protein coupled receptors modulate the etiology of AD progression. It has also been found that changes in the signaling pathways of opioid receptors increased the expression of BACE1 and γ-secretase, and is strongly correlated with abnormal production of Aβ and pathogenesis of AD. Thus, the opioid receptor family is a promising candidate for targeted drug development to treat AD. In this review, we outline the involvement and mechanisms of opioid receptor signaling modulation in Alzheimer’s Disease progression.

## Introduction

Alzheimer’s Disease (AD) is a neurodegenerative disorder that is responsible for nearly 60 to 80 percent of cases of dementia worldwide. Although the cause is still undetermined, it likely includes a combination of genetic, environmental, and lifestyle factors. Dementia has been listed as the fifth largest cause of death across the globe by the World Health Organization. A current total of 50 million people with dementia globally was estimated by AD International and is projected to increase to 152 million by 2050. The number of AD patients in the USA, which is presently 5 million, is estimated to increase to 16 million by 2050, further increasing the personal and socioeconomic burden of this disease. Every 3 seconds a patient develops dementia and the current estimate of treatment costs of dementia is currently $1 trillion USD, which is projected to double by the year 2030. In the majority of sporadic cases of AD, age plays a major role, and with an aging population worldwide, cases of AD are set to rise dramatically over the coming decades (Alzheimer’s Association, 2013; Reynolds, 2019).

**FIGURE 1:**
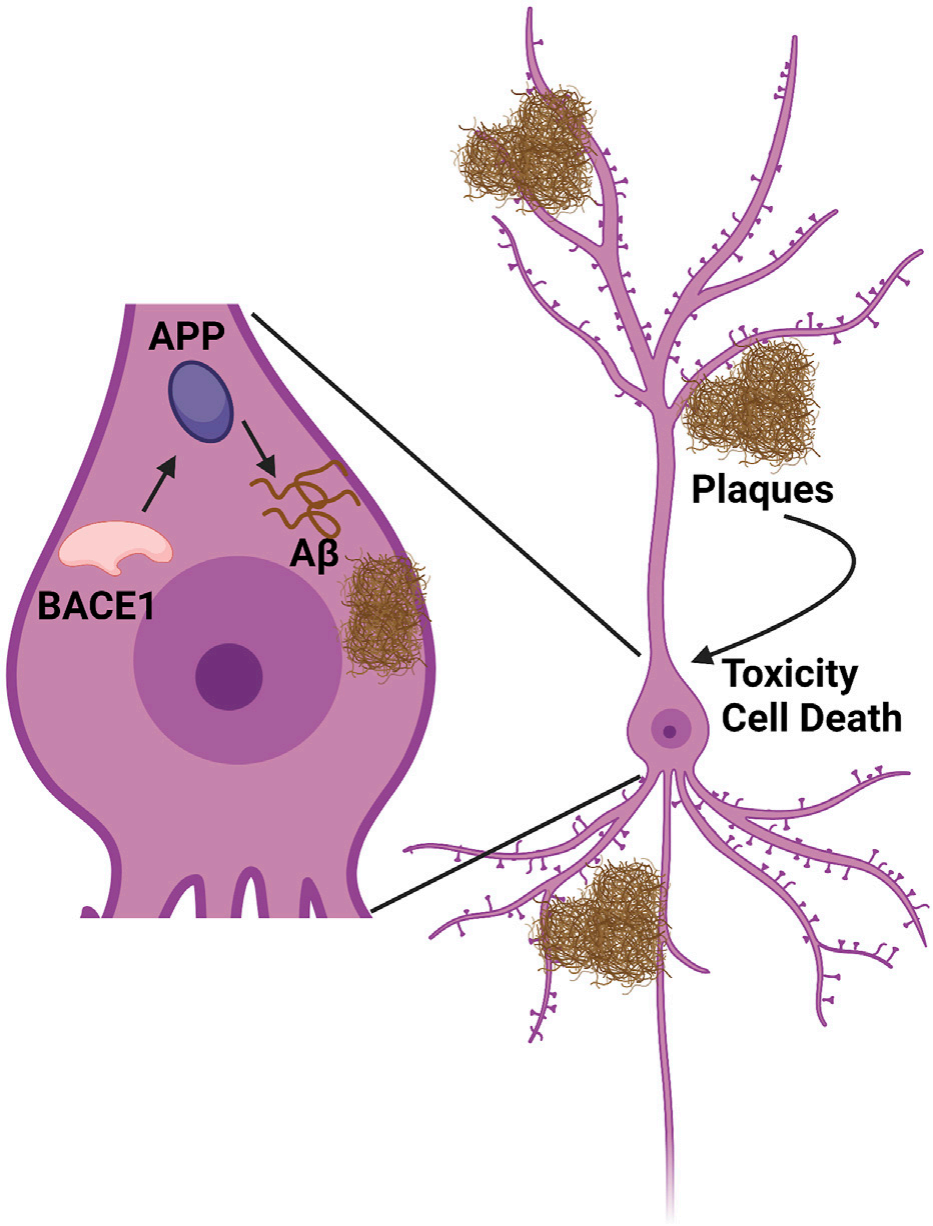
Amyloid synthesis cascade pathway. BACE1 cleaves APP to produce Aβ peptide. This peptide accumulates both intracellularly and extracellularly into tangles/plaques, which in turn cause inflammation, toxicity, and cell death. Figure created using www.biorender.com.

AD is a multifactorial progressive neurodegenerative disease characterized by memory and neuronal loss, difficulties in speaking, problem solving, and other cognitive skills, along with changes in mood and behavior, which interfere with the person’s daily performance (Ballard et al., 2011). The neuropathological hallmarks of AD include extracellular accumulation of amyloid β (Aβ) protein and intracellular accumulation of neurofibrillary tangles induced by hyper-phosphorylated Tau protein. Aβ proteins are 37–43 amino acid containing peptides that are produced from amyloid precursor protein (APP) through sequential cleavage by β-site amyloid precursor protein [APP]-cleaving enzyme 1 (BACE1) and γ-secretase. Of the two cleavage products, Aβ40 and Aβ42, AD pathogenesis correlates with the production and accumulation of Aβ42, which aggregates to form β-amyloid plaques (Figure 1) (Reynolds, 2019). Aβ42 peptides are thought to be prone to aggregation and toxicity due to their longer length (Canter et al., 2016). No disease-modifying therapies have been approved for the treatment of AD and available treatments are for symptom management with limited disease-modifying efficacy. There are many putative susceptibility risk genes for AD that have been reported, such as Apolipoprotein E (ApoE) (Corder et al., 1993), Glycogen Synthase Kinase 3-β (GSK3β) (Kwok et al., 2008; Hernández et al., 2009), Dual-Specificity Tyrosine-Regulated Kinase 1A (DYRK1A) (Kimura et al., 2007), Tau (Myers et al., 2005; Caffrey and Wade-Martins, 2007), Translocase of Outer Mitochondrial Membrane 40 Homolog (TOMM40) (Chiba-Falek et al., 2018), and Phosphatidylinositol Binding Clathrin Assembly Protein (PICALM) (Mercorio et al., 2018). Apart from these listed genetics risk factors, the APOE*ε4 allele has been shown to be responsible for Aβ accumulation and is considered the strongest confirmed genetic risk factor for early and late onset AD, while the ε2 allele is considered protective. (Corder et al., 1993; Strittmatter et al., 1993; Morris et al., 2011).

ApoE a lipid binding protein, is synthesized by astrocytes in the central nervous system (CNS) and synthesized cholesterol is transported to neurons through ApoE receptors. ApoE is polymorphic with three major alleles, namely, APOE*ε2, APOE*ε3, and APOE*ε4. People with two APOE*ε4 alleles are at a 15 times higher risk of AD as compared to APOE*ε3 carriers, and APOE*ε2 allele carriers have protection against AD. Notably, the APOE*ε3 is considered to be the most common allele and is believed to play a neutral role in the disease - neither decreasing nor increasing risk.

ApoE proteins influence with varying ability Aβ clearance from the brain, with ApoE2 being the most effective and ApoE4 the least effective (Liu et al., 2013). The APOE*ε4 allele is consistently linked to abnormal Aβ aggregation and predicts longitudinal Aβ accumulation in plaque-free elderly individuals without dementia. Conversely, APOE*ε2 carriers are protected against longitudinal Aβ accumulation (Lim et al., 2017). Unfortunately, it is evident that women are more prone to develop AD and other dementias than men. The female population with dementia is higher than males, and of the total 5.6 million AD patients, nearly 3.5 million are women and 2.1 million are men. In support of this, several studies reported that the APOE*ε4 genotype is more strongly associated with dementia in women as compared to men (Farrer et al., 1997). Together, these studies highlight the central role of the secretases and ApoE systems in regulating AD pathogenesis, and suggest that new approaches to therapeutically target these systems could be effective in modifying disease pathogenesis of AD.

## Introduction to the opioid receptor family

In the early 1990s, three important opioid receptor family members were identified and cloned after decades of investigation, namely, the μ (mu-opioid receptor-MOR), κ (kappa-opioid receptor-KOR), and δ (delta-opioid receptor-DOR) opioid receptors. In 1994, another opioid receptor was discovered, the nociceptin/orphanin FQ receptor (NOP), also called the opioid receptor-like orphan receptor (ORL) (Xia, 2015; Vaidya et al., 2018). The opioid receptor family modulates important physiological processes, including analgesia, stress response, immune response, and neuroendocrine function. The roles, locations, and functions of the opioid receptor family members are summarized in Table 1. The expression of opioid receptors and peptides are found in regions vulnerable to AD pathology in the CNS, including regions of the brain like the hippocampus and cortex, which are important for cognition and are affected heavily by AD. The opioid receptors play important roles in synaptic activation, learning and memory. Administration of opioid antagonists has been found to significantly improve the memory performance of rats (Gallagher, 1985). These antagonists and similar drugs discussed here are summarized in Table 2. However, later studies have failed to support the efficacy of the non-selective antagonists naloxone or naltrexone in improving AD symptoms (Sunderland et al., 1986). Despite this, there is a strong overlap in the distribution of opioid receptors and amyloid plaque location in AD patients, leading the field to postulate a role of these three opioid receptors in the pathology of AD. Opioid receptor knockout mouse studies further demonstrated a potential regulatory role in AD due to the distinct and opposing roles of opioid receptors in modulating different animal behaviors important in AD, such as locomotion, anxiety, depressive behavior, or alcohol intake (Kieffer and Gavériaux-Ruff, 2002).

**TABLE 1:** Opioid group receptor subtypes, function & location in brain.

Receptor subtypes	Function	Location	Mechanism of action	References
δ (delta)	analgesia; memory; anxiety and mood; modulation of hormone and neurotransmitter release	Hippocampus, spinal cord, dorsal root ganglia, periphery, hypothalamus, brain stem	Gα i/o	Mafi et al. (2021), Tanguturi et al. (2022)
μ (mu)	Supraspinal and spinal analgesia; respiratory depression	Cortex, limbic system, brain stem, periphery, reward circuitry (ventral tegmental area, striatum)	Gα i/o	
	endocrine activity; sedation; slowed gastrointestinal transit			Nam et al. (2021)
	hormone modulation and release of neurotransmitter			
κ (kappa)	Supraspinal and spinal analgesia; motor control; stress response; mood; psychotomimetic effects	Periphery, dorsal root ganglion, spinal cord, reward circuitry (striatum), amygdala	Gα i/o	Al-Hasani and Bruchas (2011), Stefanucci et al. (2023)

**TABLE 2:** Opioid agonist and antagonist drugs.

Agonists	Antagonists
Morphine Pathan and Williams (2012)	Naloxone Stefanucci et al. (2022)
Alfentanil	Naltrexone Comer et al. (2006)
Codeine Yazdy et al. (2015)	Nalmefene Krieter et al. (2019)
Dextromethorphan Chen et al. (2005)	Naltrindole Granier et al. (2012)
Dextropropoxyphene Barkin et al. (2006)	
Fentanyl Moussawi et al. (2020); Lim et al. (2022)	
Methadone Strain et al. (1999)	
Pethidine O’Connor et al. (2000); Listos et al. (2019)	
SNC80 Metcalf et al. (2012); Sakamoto et al. (2021)	
DPDPE Stefanucci et al. (2017)	
DADLE Förster et al. (2007)	

Opioid receptors are members of a superfamily of 7 transmembrane spanning (7TM) G protein-coupled receptors (GPCRs). So far, almost 370 non-olfactory GPCRs have been identified, out of which nearly 90% are present in the brain, playing crucial roles in mood, cognition, pain, appetite, and synaptic transmission (Vassilatis et al., 2003). Opioid receptors are also found in the nervous system, lungs, heart, liver, and the gastrointestinal and reproductive tracts (Jutkiewicz, 2018). GPCRs are involved in multiple neurotransmitter systems that are associated with AD; glutamatergic, serotonergic, adrenergic and peptidergic pathways in particular are dysregulated in this neurodegenerative disorder and are impacted by opioid receptor activity (Thathiah and De Strooper, 2011). Targeting these systems might protect against disease progression by modulating the formation of Aβ plaques or aberrant signaling following plaque formation (Thathiah and De Strooper, 2011). Activation of opioid receptors occurs by endogenous peptides as well as opioid drugs such as morphine, which are administered exogenously. These are not only effective analgesics, but also cause side effects such as addiction and are categorized as drugs of abuse, providing a note of caution in exploiting this receptor family (Satoh et al., 2000a).

There are many notable similarities in the primary structures of MOR, DOR and KOR along with their function and mechanisms of intracellular signaling (SKARPHEDINSSON and THOREN, 1988). Adding further complexity, both homomeric and heteromeric complex formation between opioid receptors and non-opioid receptors can lead to modification of the response to a particular opioid ligand (Satoh et al., 2000; Wei and Loh, 2002; Pasternak, 2004; Ananthan, 2008). A pharmacological response generated by action of opioid ligands might be due to interaction with different opioid receptor complexes. On the other hand, synthetic opioid peptides and alkaloids are very selective for MOR, DOR, and KOR, and have been explored largely for defining pharmacological properties of isolated opioid receptors. This means that potential unexplored complexities of opioid receptor function could impact their activity in AD.

GPCRs serve as versatile targets from the perspective of drug discovery. Various drugs which target GPCRs can act either as agonists or as antagonists for the signaling of G protein. A conformational change is promoted after binding of agonist to a GPCR resulting in the activation of receptor-associated heterotrimeric G proteins and further downstream signaling pathways. However, a small family of multifunctional GPCR regulatory or adaptor proteins known as the β-arrestins, which have an almost universal role in facilitating traditional GPCR desensitization, are also capable of initiating distinct, independent signaling events (DeWire et al., 2007). In AD pathogenesis, GPCRs are found to be associated with various stages of APP proteolysis, which includes modulation of APP processing by the α-, β- and γ-secretases and regulating degradation of Aβ and Aβ-mediated toxicity (Thathiah and De Strooper, 2011; Wisely et al., 2014). Studies have shown that GPCRs can bind to β-secretase and γ-secretase, which are key enzymes in the hydrolytic processing of APP (Liu X. et al., 2013; Nelson and Sheng, 2013; Thathiah et al., 2013). Unfortunately, the signaling mechanisms, which mediate these effects have not been completely explored and the regulation of the γ-secretase complex by GPCRs as hypothesized remains unexplained.

The opioid receptors are involved in learning and memory and are dysregulated in specific regions of the AD brain (Mathieu-Kia et al., 2001). Recent studies suggest that these GPCRs and one of their ligands, enkephalin, are involved in modulation of β-secretase and subsequent Aβ generation (Thathiah and De Strooper, 2011).

## The role of opioid receptors in modulating Alzheimer’s disease

Opioid receptors have a modulatory effect on the regulation of neurotransmitters such as acetylcholine, norepinephrine, GABA, glutamate, and serotonin, which have been implicated in the pathogenesis of AD. A close relationship exists between Aβ generation and the opioid system, as dysfunction of the opioid receptors causes retardation of endocytosis and degradation of the BACE1 enzyme; this enzyme is required for generating monomeric forms of Aβ, including Aβ42, which further aggregates into bioactive conformational species and is responsible for initiation of toxicity in AD (Hampel et al., 2020). This directly suggests that dysfunctional opioid receptors can contribute to AD pathology. It has also been reported that alteration in the signaling pathway of opioid receptors is strongly correlated with abnormal production of Aβ and pathogenesis of AD (Sunderland et al., 1986).

No approved disease-modifying therapies are available for AD and the few agents available for symptomatic treatment are less effective. Various efforts have taken place for prevention or elimination of beta-amyloid plaques, including targeting the BACE1 and γ-secretase enzymes, but unfortunately none of these strategies have been clinically successful. This suggests that new approaches to inhibiting the production of Aβ in the first place may be needed (Scheltens et al., 2016).

Studies have reported that indirectly modulating the function of enzymes *via* modulating GPCRs could serve as a novel strategy for reducing the production of Aβ peptide with less side effects. Among GPCRs that influence amyloidogenesis, the DOR has been shown to play an important role in the trafficking and function of BACE1, γ-secretase, and the production of Aβ peptide. Studies have reported that DOR levels in certain specific regions of AD brains were elevated compared with non-AD brains. These notable areas included the frontal cortex, caudate, and hippocampus (Hiller et al., 1987; Mathieu-Kia et al., 2001). It has been suggested that the DOR along with the β2 adrenergic receptor (β2AR) leads to promotion of cleavage of the APP C-terminal fragment mediated by γ-secretase, once it is generated by β-secretase (Ni et al., 2006a). The DOR has shown a strong modulatory role in AD by showing its effect on secretase activity after stimulation with its agonist for 30 min, which enhanced BACE1 and γ-secretase activities to 143% and 156% respectively. This was reported using a fluorogenic substrate assay using a HEK293T cell line overexpressed with DOR, while the activity of α-secretase was unaltered (Teng et al., 2010). Research on *postmortem* brains of AD patients has shown that MOR, DOR, and KOR are differentially altered in distinct brain areas (Mathieu-Kia et al., 2001c). DOR binding is decreased in the amygdala and ventral putamen, and MOR binding is decreased in the hippocampus and subiculum (Mathieu-Kia et al., 2001) of *postmortem* brain samples from patients with AD. The levels of leu-enkephalin and dynorphin A (the endogenous opioid peptides for DOR and KOR, respectively) were increased in the frontal cortex of patients with AD in this same study. Elevation of the opioid precursors pre-proenkephalin and met-enkephalin was found to contribute to the cognitive and behavioral decline in a transgenic mouse model of AD, suggesting the involvement of DOR in AD pathology (Meilandt et al., 2008a). Early studies on possible treatment of AD using the non-selective opioid antagonists naloxone and naltrexone did not find them to be efficacious. However, more recent studies focusing on specific subtypes of opioid receptor have revealed that the DOR in particular plays a significant role in AD pathology (Teng et al., 2010; Liu et al., 2013).

**FIGURE 2:**
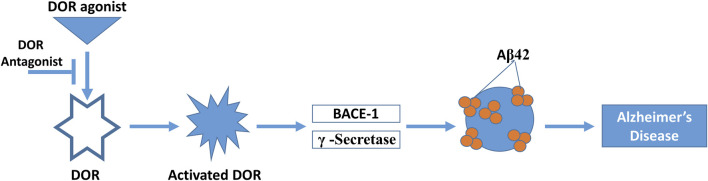
Interaction of the DOR with Alzheimer’s pathology. DOR is activated by DOR agonist, which in turn activates BACE1 and γ-secretase activity. This produces Aβ42 protein aggregates, producing at least in part Alzheimer’s disease pathology. This process can be reversed by DOR antagonist treatment.

Studies have reported that agonist-induced activation of DOR has been shown to increase BACE1 and γ-secretase activity *in vitro* in cells and *in vivo* in an AD mouse model leading to increased production of Aβ peptide (Figure 2) (Ni et al., 2006; Teng et al., 2010). Mechanistic studies revealed that DOR forms a complex with β- and γ-secretases and that the DOR mediates co-endocytic sorting of this complex into late endosomal/lysosomal (LEL) compartments, in which the generation of Aβ takes place. DOR antagonism using naltrindole substantially reversed AD pathology caused by APP/Presenilin overexpression in mice, including memory deficits, reactive glia formation, Aβ production, and BACE1/γ-secretase activity in the brain (Teng et al., 2010). Similarly, it has been observed *in vivo* in an AD mouse model that DOR knockdown resulted in reduced accumulation of Aβ40 in the hippocampus. However, there was no effect on the more hydrophobic (and more toxic) Aβ42 (Teng et al., 2010). No effect was observed with administration of MOR antagonist on the generation of Aβ or amyloid plaque formation, and MOR antagonist was not able to reverse the learning and memory deficiency of the AD mouse model (Teng et al., 2010), although another group reported improved spatial memory retention in this transgenic AD mouse model with MOR antagonist treatment (Meilandt et al., 2008). Importantly, neither activation of DOR *in vitro* nor blockage of DOR *in vivo* affected the processing of Notch, N-cadherin, or APLP-1 by either BACE1 or γ-secretase, thus providing direct evidence that antagonism of DOR specifically blocks the amyloidogenic pathway and efficaciously prevents AD progression in mice (Teng et al., 2010). However, DOR antagonists have not yet been explored as therapeutic agents for AD in humans.

In contrast to the above work, a recent study reported very different roles of DOR and MOR in the regulation and activity of BACE1 expression, highlighting the possible neuroprotective role of DOR against AD injury. Activation of DOR with its specific agonist UFP-512 substantially decreased BACE1 expression and its activity in a highly differentiated rat PC-12 cell line with imitated AD injury, whereas DOR antagonism with naltrindole reversed the UFP-512 effects while causing an enhancement of BACE1 expression and activity along with Aβ42 production under physiological conditions (Antonino et al., 2022). Knocking-down DOR *in vivo* increased BACE1 protein expression and its activity for APP processing, leading to a significant increase in Aβ42 production (Antonino et al., 2022). A different study found an association between opioid abuse and combined neuroinflammation and hyperphosphorylated tau in patients, which may confirm a pro-AD role for MOR activation (Anthony et al., 2010). Considering the opposite roles of MOR and DOR in activating BACE1 expression, there is a possibility to come up with a novel strategy against AD by differentially targeting DOR and MOR (Xu et al., 2020). Overall, these contrasting results of the studies reported above highlight the complexity of opioid activity in AD and highlights the need for further study to work out these differences.

In a genetic meta-analysis of AD patient cohorts, Sarajarvi and others found that a heterozygous DOR-Phe27Cys mutation increased the risk of AD, especially in late-stage AD patient *postmortem* brain samples in which there was a significant upregulation of BACE1 and γ-secretase activities (Sarajarvi et al., 2015). This is an important finding since approximately 25% of AD patients are heterozygous for the DOR-Phe27Cys variation and could potentially be used in patient selection and stratification for clinical studies using DOR antagonists as novel AD therapeutics. These observations clearly suggest that antagonism of DOR could be a novel therapeutic approach for the treatment of AD (Sarajärvi et al., 2015). In contrast to antagonism of DOR, agonist activation of MOR has also been reported to have salutary activity against AD through several mechanisms (Cui et al., 2011; Wang et al., 2015a; Dhull and Kumar, 2018). These studies suggest that selective antagonists of DOR and mixed DOR-antagonist/MOR-agonists could potentially emerge as a new therapeutic strategy against AD with fewer side effects (Figure 2).

Several biochemical and pharmacological studies using MOR and DOR ligands gave an early indication of physical and functional interactions between the two receptors (Starke et al., 1990). The MOR and DOR are present in pain modulating regions of the CNS on overlapping populations of neurons. It has also been shown that both MOR and DOR are present within the same functionally distinct heterodimeric or hetero-oligomeric complexes (George et al., 2000; Gomes et al., 2000; Levac et al., 2002; Fan et al., 2005). The physiological and pharmacological significance of MOR/DOR interactions have been substantiated by recent studies using opioid receptor gene knockout animals (Kieffer, 1999). Both MOR and DOR have an intermodulatory effect suggesting that ligands having a mixed interaction profile at both the receptors or at specific dimer pairs could serve as a novel therapeutic approach for the treatment of AD.

The KOR plays an important role in cognitive and learning functions and may also be involved in modulating AD pathology. The KOR (k1 and k2 variants) is among the most abundant brain opioid receptors. In the human brain, KOR has a wide and distinct distribution in the neocortex, striatum, thalamus, amygdala, and hippocampus (Simonin et al., 1995; Hiller and Fan, 1996) and is implicated in the pathophysiology of depression, anxiety, and alcoholism (McLaughlin et al., 2003; Tejeda et al., 2012). KOR activation was associated with stress-related dysmnesia, and these receptors could regulate glutamate neurotransmission and affect synaptic plasticity underlying memory formation. An early study showed evidence of increased kappa binding sites of AD brains at autopsy, indicating that KOR may also be involved in AD and other disorders, such as epilepsy and Tourette’s syndrome (Loacker et al., 2007; Cai and Ratka, 2012).

AD is a complex disease affected by both environmental and genetic factors (Xu et al., 2013). Epigenetics has emerged as an intermediate between environmental and genetic factors which could impact AD (Cai and Ratka, 2012). As an important component of epigenetics, genes with significantly changed DNA methylation have been found in AD patients (Van den Hove et al., 2014). Targeting epigenetic DNA methylation could be a therapeutic target for neurodegenerative disease such as Huntington’s disease and AD (Chang et al., 2014). Studies conducted on methylation of opioid receptor genes found that promoter DNA methylation may be dysregulated and thus may play a role in disease pathology (Sun et al., 2015). Several studies found that increased methylation levels of the four opioid receptor genes *OPRM1*, *OPRL1*, *OPRK1* and *OPRD1* indicate the epigenetic involvement of the opioid system in AD patients compared with healthy subjects (Xu et al., 2018). The above findings also suggest that opioid receptor genes could be used as potential methylation biomarkers for the diagnosis of AD (Ji et al., 2015a; Sun et al., 2015). A higher level of DNA methylation in the KOR (*OPRK1*) promoter CpG site was observed in patients with AD as compared to controls and this high methylation of *OPRK1* could contribute to the risk of AD through its downregulation of gene expression (Ji et al., 2015).

Lastly, mechanistic/mammalian target of rapamycin (mTOR) has a very important role in neuronal plasticity, learning and memory and has a close association with many neurodevelopmental and neuropsychiatric disorders (Costa-Mattioli and Monteggia, 2013). Studies have reported that MOR activation attenuated Aβ oligomer-induced neurotoxicity through mTOR signaling. It may provide new insight into the pathological process and useful strategy for therapeutic interventions against Aβ neurotoxicity by targeting this mTOR/MOR relationship (Wang et al., 2015).

## Future perspective

The studies above emphasize the importance of opioid receptors in AD modulation; however, the area needs to be explored further for better understanding. AD is a complex neurological disorder and currently there are no approved disease modifying therapies available. Efforts are underway to eliminate β-amyloid plaques through inhibiting two notable enzymes such as BACE1 and the γ-secretase enzyme. Nevertheless, these approaches have not yet achieved clinical success. Studies have recommended that indirect modulation of the function of these enzymes *via* opioid receptors be pursued to achieve reduction in Aβ pathology and identify lead compounds for further development as candidate AD therapeutics.
